# By-Laws of the Section of Meteorology, Medical Topography, and Epidemics

**Published:** 1866-07

**Authors:** N. S. Davis

**Affiliations:** Secretary


*By-Laws of the Section of Meteorology, Medical Topography, and Epidemics.*

1st. The officers of the section shall consist of a President and Secretary, who shall continue their office one year, and until their successors are elected.

2d. The Secretary shall keep a fair record of the doings of the section, with an abstract of all discussions of papers and questions acted upon by the section, and report the same to the Association, or to the Permanent Secretary of the Association, within thirty days after the adjournment of each annual meeting.

3d. When any report, paper, or question is read to the section it shall be subject to a full discussion, but no member shall speak more than once on the same subject, until all others who wish to speak have been heard.

4th. Every report or paper referred to the section shall receive a sufficient examination, to determine fully its contents and merits, before it can be recommended for reference to the Committee of Publication. And whenever reports or papers are presented of such length and nature that the section cannot give them the necessary examination during the limited time of its annual sessions, it shall refer them to a sub-committee, with instructions to complete their examination, and report the result to the Permanent Secretary of the Association, within thirty days after the adjournment of the annual meeting.

5th. It shall be the duty of the section to select such topics for original investigation, and refer them to special committees, as will be best calculated to increase our knowledge of those departments of medical science placed in charge of the section.

On motion, the report of the committee was accepted, and the foregoing rules adopted unanimously.

The Secretary stated that a paper had been referred to the section for consideration, on the "Etiological and Pathological relations of Epidemic Erysipelas, Spotted Fever, or Cerebro-Spinal Meningitis, and Diphtheria," by Dr. N. S. Davis, of Chicago, Illinois.

The reading of the paper having been called for, it was read in full by the author. It was listened to with interest and attention; and the allusions in it to the etiology of epidemics generally, led to a very interesting discussion of the question, of how far *local meteorological and sanitary* conditions influence the origin and spread of epidemic cholera?

Dr. Woodward, of Fort Wayne, Indiana, said that sporadic cases of genuine spasmodic cholera occurred in his locality every summer. He stated that two cases came under his observation during the summer of 1865, presenting all the phenomena of true cholera, as perfectly as he had ever observed in the midst of an epidemic of that disease. He thought epidemics usually originated from an exaggeration of the same local influences that gave rise to sporadic cases, as suggested in the paper just read.

Dr. Worthington Hooker, of New Haven, Connecticut, stated that he had seen cases of real cholera during its epidemic prevalence, so perfectly disconnected from all other cases, that contagion or communication was not possible. He could attribute such cases to no other than local causes. He stated that there appeared to be three theories in relation to the origin and spread of cholera. The first was that which made it depend entirely on a specific poison of a contagious and portable character. The second made the disease depend upon an infectious poison or miasm, capable of acting only when there are special local qualities of the atmosphere favorable for its action or increase. The third attributes its origin and spread exclusively to local meteorological and sanitary influences. His own convictions were, that in periods when the cholera prevails as an epidemic, certain unexplained telluric influences are added to local causes, which render the latter more active, and gives to the disease greater tendency to spread from place to place with some regularity.

Dr. Stockwell, of Michigan, gave an interesting account of the prevalence of epidemic cholera on one side of the St. Clair river, during the summer of 1854, in a limited district, while the other side of the river and surrounding places were exempt, though intercourse remained entirely unobstructed. In the district where the disease prevailed, the soil was level, and immediately underlaid with tenaceous clay, thereby preventing ready escape of the surface water, and exerting an influence on the electrical condition of the atmosphere. The soil on the other side of the river was sandy and porous, allowing of the most rapid percloration of water.

Dr. N. S. Davis, of Chicago, remarked that we were yet without the data necessary for determining with certainty either the origin or mode of spread of cholera. If we accept one series of facts, and confine our attention to them, we shall be led directly to the doctrine of contagion and portability. If we accept another series of facts, equally well established, we shall be just as certainly led to the conclusion that cholera arises from local causes. But if we examine critically the circumstances claimed as facts in either series, it will be found that a large proportion of them have been so imperfectly observed, or recorded in such careless general terms, that they are of very little value. He had participated actively in the study and treatment of five different epidemics of cholera, and so far as his own observations were concerned, they led him directly to the conviction that the disease neither travels from country to country, nor propagates itself by contagious virus, or infectious dejections. He mentioned many facts of interest, but claimed that he could not settle definitely the origin of epidemics until more systematic meteorological and sanitary records were kept from year to year, in connection with equally exact records of the prevalence and specific character of diseases. When this has been done long enough to cover the periodical return of two or three epidemics, we shall be able to command all the elements necessary for a comparison, *etiologically*, of epidemic seasons, with those which precede and follow.

Then, and not until then, can we, with confidence, deduce such conclusions as should guide, both the profession and the municipal authorities, in the adoption of sanitary laws.

On motion of Dr. Wilson, the paper that had been read to the section, was referred to the Committee of Publication.

The following resolution was offered by Dr. Davis, and adopted, viz.:—

*Resolved*, that the secretary of this section be requested to enter into correspondence with the members of the Committee on Meteorology and Epidemics in the several States, and such other persons as he may think proper, for the purpose of establishing a uniform system of meteorological and sanitary records, embracing the thermometric, barometric, hygrometric, electric, and ozonic conditions of the atmosphere; the topography; and the sanitary conditions; in connection with a coincident record of the kind, special character, and extent of the prevalent diseases, at representative points throughout the whole country.

On motion the section adjourned.

N. S. Davis, Secretary.

